# IL-1β drives SARS-CoV-2-induced disease independently of the inflammasome and pyroptosis signalling

**DOI:** 10.1038/s41418-025-01459-x

**Published:** 2025-02-28

**Authors:** Stefanie M. Bader, Lena Scherer, Jan Schaefer, James P. Cooney, Liana Mackiewicz, Merle Dayton, Smitha Rose Georgy, Kathryn C. Davidson, Cody C. Allison, Marco J. Herold, Andreas Strasser, Marc Pellegrini, Marcel Doerflinger

**Affiliations:** 1 The Walter and Eliza Hall Institute of Medical Research (WEHI), Parkville, VIC 3052 Australia; 2 Department of Medical Biology, University of Melbourne, Melbourne, VIC Australia; 3 Anatomic Pathology—Veterinary Biosciences, Melbourne Veterinary School, University of Melbourne, Werribee, VIC 3030 Australia; 4 Olivia Newton-John Cancer Research Institute, Heidelberg, VIC Australia; 5 School of Cancer Medicine, La Trobe University, Bundoora, VIC Australia; 6 Centenary Institute and University of Technology Sydney, Faculty of Science, School of Life Sciences, Sydney, NSW Australia

**Keywords:** Preclinical research, Cell death and immune response, Infectious diseases, Inflammasome

## Abstract

Excessive inflammation and cytokine release are hallmarks of severe COVID-19. Certain programmed cell death processes can drive inflammation, however, their role in the pathogenesis of severe COVID-19 is unclear. Pyroptosis is a pro-inflammatory form of regulated cell death initiated by inflammasomes and executed by the pore-forming protein gasdermin D (GSDMD). Using an established mouse adapted SARS-CoV-2 virus and a panel of gene-targeted mice we found that deletion of the inflammasome (NLRP1/3 and the adaptor ASC) and pore forming proteins involved in pyroptosis (GSDMA/C/D/E) only marginally reduced IL-1β levels and did not impact disease outcome or viral loads. Furthermore, we found that SARS-CoV-2 infection did not trigger GSDMD activation in mouse lungs. Finally, we did not observe any difference between WT animals and mice with compound deficiencies in the pro-inflammatory initiator caspases (*C1/11/12*^*−/−*^). This indicates that the classical canonical and non-canonical pro-inflammatory caspases known to process and activate pro-IL-1β, pro-IL-18 and GSDMD do not substantially contribute to SARS-CoV-2 pathogenesis. However, the loss of IL-1β, but not the absence of IL-18, ameliorated disease and enhanced survival in SARS-CoV-2 infected animals compared to wildtype mice. Collectively, these findings demonstrate that IL-1β is an important factor contributing to severe SARS-CoV-2 disease, but its release was largely independent of inflammasome and pyroptotic pathways.

## Introduction

SARS-CoV-2 causes a spectrum of disease, ranging from mild resolving illness to severe COVID-19 and mortality. The host factors contributing to the spectrum of SARS-CoV-2 associated disease are yet to be characterised. Unravelling the molecular mechanisms that contribute to the dysregulation and aberrant activation of the immune system following SARS-CoV-2 infection is paramount for formulating effective strategies to mitigate the morbidity and mortality caused by this pandemic virus.

Emerging evidence suggests that inflammation and certain programmed (regulated) cell death processes play pivotal roles in the pathogenesis of COVID-19 (reviewed in [1–3]). Lytic forms of regulated cell death, such as necroptosis and pyroptosis, have been linked to dysregulation of the immune system associated with COVID-19 through the release of damage associated molecular patterns (DAMPs), cytokines and immune activation and inflammation linked to cell membrane disruption [4–10]. Pyroptosis is a distinct form of programmed (regulated) cell death characterised by features of necrosis and an inflammatory response. It is triggered by the activation of inflammatory caspases-1 (human and mouse), -4 (human), -5 (human), and/or -11 (mouse) [11], which proteolytically activate the pyroptotic pore forming effector protein gasdermin D (GSDMD) [12]. The activation of the specific caspases associated with pyroptosis is regulated by inflammasomes in response to certain infections and host-derived proteins and crystals [13, 14]. The inflammasome sensors nucleotide oligomerisation domain (NOD)-like, leucine-rich repeat (LRR) receptor, absent in melanoma 2 (AIM2), and pyrin require the adaptor protein apoptosis-associated speck-like protein containing a CARD (ASC) to form a functional complex to activate caspase-1. Conversely, NLRC4 and NLRP1b inflammasomes can directly bind to and activate caspase-1 without ASC. In addition to proteolytically activating GSDMD, caspase-1 also cleaves the pro-inflammatory cytokines pro-interleukin-1β (IL-1β) and pro-interleukin-18 (IL-18) into their bioactive forms that can be secreted by cells. Upon assembly, the negatively charged GSDMD pore allows the rapid release of these proteolytically processed pro-inflammatory cytokines into the extracellular milieu [11, 15]. The secreted bioactive form of IL-1β is a pleiotropic cytokine that orchestrates a complex network of downstream mediators, amplifying inflammatory responses, promoting the recruitment of innate immune cells, and modulating the activity of adaptive immune cells [16]. Excessive secretion of IL-1β is a well-established contributor to the pathogenesis of various inflammatory diseases, including pulmonary infections caused by influenza virus [17]. Mature IL-18 is involved in the production of interferon-gamma (IFN-γ) and promotes Th1 and Th2 T cell immune responses [18, 19].

Necroptosis is another lytic form of programmed (regulated) cell death that triggers the release of DAMPs to promote immune activation, cytokine release and inflammation [12, 20]. Necroptosis can occur downstream of death receptor signalling, such as ligation of TNF receptor 1 (TNFR1) [21, 22], through the activation of cytoplasmic nucleic acid sensor Z-DNA binding protein 1 (ZBP1) [21, 22] and downstream of Toll-like receptors (TLR) through the stimulation of Toll/IL-1 receptor domain-containing adapter-inducing interferon-β (TRIF) [23, 24]. These signals lead to activation of the kinase RIPK3, typically in the absence of proteolytically active caspase-8 [25]. Once activated, RIPK3 phosphorylates the pseudokinase mixed lineage kinase domain-like (MLKL) causing its oligomerisation and translocation to the plasma membrane, where it causes membrane rupture and a necrotic form of cell death [26–28].

Lytic forms of programmed (regulated) cell death have been proposed to contribute to COVID-19 pathogenesis, but genetic in vivo evidence is incomplete [3]. In our recent study utilising gene-targeted mouse models of severe COVID-19, we challenged the notion that necroptosis was a significant driver of this disease [29]. This highlighted the need for further animal studies to dissect COVID-19 pathogenesis which involves several cell types and organ systems. Here, we utilised diverse gene targeted mouse strains to investigate the relevance of lytic programmed cell death pathways and their contribution to inflammation during severe SARS-CoV-2 infection driven disease. We found that IL-1β drives SARS-CoV-2 disease severity, but surprisingly, independently of pyroptosis and the inflammasome.

## Results

### Loss of inflammasome signalling does not prevent pro-inflammatory cytokine release and disease following SARS-CoV-2 infection in mice

**Fig. 1:**
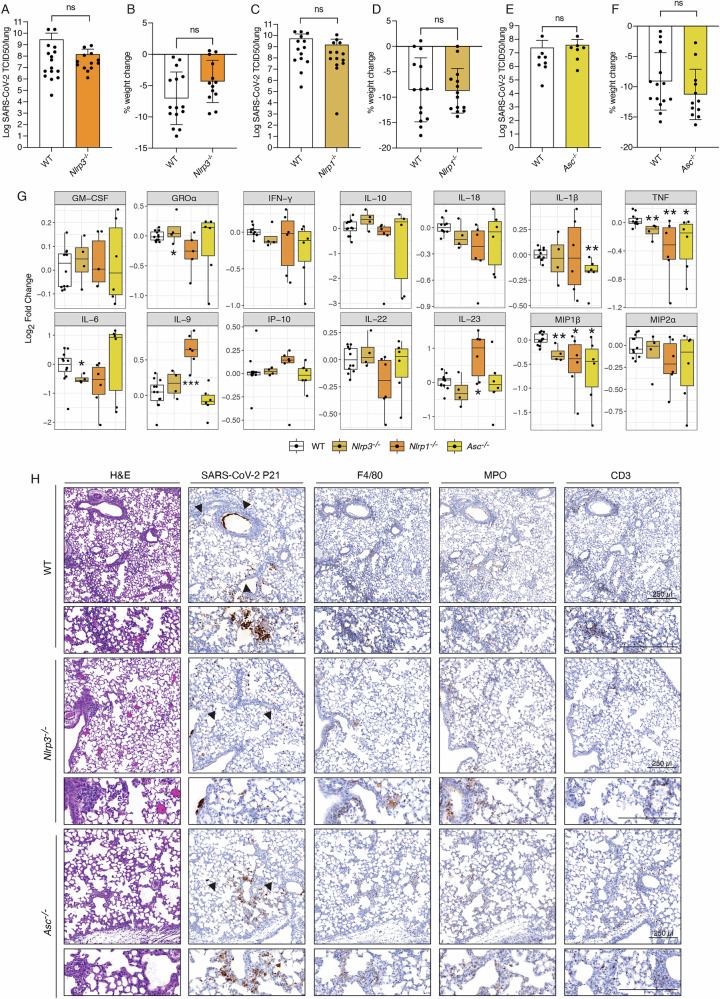
Inflammation driven by SARS-CoV-2 infection is independent of inflammasomes. WT and *Nlrp3* knockout (*Nlrp3*^*−/−*^) mice were infected with 10^4^ TCID50/mouse of SARS-CoV-2 P21 and examined at 3 days post-infection (dpi) for (**A**) lung viral burden by TCID50 assay and (**B**) percent weight change compared to initial weight. Results are pooled from 2 independent experiments (*n* = 13–17 mice per genotype). WT and *Nlrp1* knockout mice (*Nlrp1*^*–/–*^) were challenged intranasally with 10^4^ TCID50/mouse of SARS-CoV-2 P21 and examined 3 days post-infection (dpi) for (**C**) lung viral burden using a TCID50 assay and (**D**) percent weight change compared to initial weight (*n* = 14 mice per genotype). WT and *Asc* knockout (*Asc*^*–/–*^) mice were infected with 10^4^ TCID50/mouse of SARS-CoV-2 P21 and monitored at 3 dpi for (**E**) lung viral burden by using a TCID50 assay (*n* = 7–8 mice per genotype) and (**F**) percent weight change compared to initial weight. Results are pooled from 2 independent experiments (*n* = 12–15 mice per genotype). **G** Levels of cytokines and chemokines measured by ELISA of lung homogenates of WT and the indicated knockout animals 3 days post SARS-CoV-2 P21 infection (*n* = 5–10 mice per genotype). **H** Representative images of haematoxylin and eosin (H&E) and immunohistochemistry (IHC) stained lung sections testing for SARS-CoV-2 nucleocapsid, F4/80 (marker of macrophages), MPO (marker of neutrophils) and CD3 (marker of T cells). Mice of the indicated genotypes were challenged intranasally with 10^4^ TCID50/mouse of SARS-CoV-2 P21 and lungs were collected and fixed for histological analysis at 3 dpi. Histological images are representative of at least 3 animals per genotype. Black arrows point to exemplary SARS-CoV-2-positive cells. Scale bar = 250 µm. Unpaired two-tailed Student’s *t* test after log_10_ transformation (**A**, **E**), Mann–Whitney test (**C**), unpaired two-tailed Student’s *t* test (**B**, **D**, **F**), Wilcoxon rank-sum (**G**), statistical tests were performed. In (**G**), significance is shown relative to WT control mice (*<0.05; **p < 0.01).

To investigate the role of inflammasome signalling pathways during severe SARS-CoV-2 infection-induced disease we used a mouse adapted strain called P21 [30]. This virus strain was derived from a clinical SARS-CoV-2 isolate that was passaged 21 times in C57BL/6 mice to allow adaptations that are able to cause severe disease in WT mice, characterised by weight loss and increased levels of pro-inflammatory cytokines [30]. SARS-CoV-2 P21 has been extensively characterised, and its relevance to COVID-19 thoroughly evaluated [30]. It has been validated and widely utilised in studies to elucidate host biology [29, 31] and to assess the efficacy of vaccines and therapeutic agents [32–35]. During SARS-CoV-2 infection, activation of the NOD-like receptor family pyrin domain-containing 3 (NLRP3) inflammasome pathway is thought to contribute to the release of bioactive IL-1β and IL-18, cytokines associated with severe COVID-19 [6, 7, 36, 37]. To explore this hypothesis, we infected NLRP3 deficient mice (*Nlrp3*^*−/−*^) with P21. Interestingly, three days post infection (dpi), *Nlrp3*^*–/–*^ mice displayed similar viral burdens to WT controls and did not show changes in weight loss, an established marker of disease severity in P21-infected mice at this time point (Fig. 1A, B). NLRP1 is also a potent activator of inflammation [38, 39]. However, we observed that severe disease and lung viral burdens caused by SARS-CoV-2 infection were not affected by deletion of NLRP1 in mice **(**Fig. 1C–D**)**. Many of the inflammasomes, including NLRP1, NLRP3, AIM2, are dependent on the adapter protein ASC for caspase-1 activation [19, 40–42]. Surprisingly, ASC deficient mice (*Asc*^*–/–*^) exhibited viral burdens and weight loss similar to infected WT animals (Fig. 1E, F). This demonstrates that ASC dependent inflammasomes are not essential for SARS-CoV-2 driven pathogenesis.

We have previously shown that infection with SARS-CoV-2 P21 causes increased production of a range of pro-inflammatory cytokines in the lungs of infected WT animals at multiple time points post-infection, including IL-1β, IL-18, IL-6, TNF, IFN-γ and others [30]. To better understand if lytic programmed cell death processes and canonical inflammasome activation contributed to this cytokine release, we next measured the levels of 25 cytokines and chemokines in lung homogenates from infected gene-targeted mice using WT animals as controls. To allow for easier comparison across different experiments and genotypes, we present cytokine data as fold-induction to normalise responses to baseline levels. Interestingly, neither of the inflammasome knockout mice we investigated displayed a reduction in IL-18, while only *Asc*^*−/−*^ animals had slightly diminished levels of IL-1β in their lungs upon SARS-CoV-2 infection (Fig. 1G). While *Nlrp3*, *Nlrp1* and *Asc* inflammasome knockout mice all showed a reduction in the levels of TNF, MIP1α and IL-17a, variable differences in the levels of other cytokines were observed, depending on the particular inflammasome deficiency (Fig. 1G and S1A). Overall, these results indicate that while NLRP1/3 and ASC do affect cytokine release during SARS-CoV-2 infection, this was not critical for disease severity (weight loss) or viral burden compared to WT mice.

To better understand disease in SARS-CoV-2 P21 infected gene-targeted animals, we compared lung histology between knockout versus WT mice at 3 dpi. Lung sections were stained with haematoxylin and eosin (H&E) and analysed by a board-certified pathologist. At 3 dpi, WT mice displayed multifocal, acute alveolitis (sometimes necrotising), multifocal pneumonia, as well as moderate to severe acute multifocal perivasculitis (Fig. 1H). Gene-targeted mice showed similar manifestations of disease with interstitial pneumonia, moderate to severe multifocal perivasculitis and acute alveolitis. Immunohistochemical (IHC) staining for SARS-CoV-2 nucleocapsid showed that the virus localised to the bronchiolar and alveolar epithelium as well as macrophages in both WT and all gene-targeted mice (Fig. 1H). Staining for myeloperoxidase (MPO), CD3 and F4/80 revealed a similar number of myeloid and T cell infiltrates in WT and knockout mice tested (Fig. 1H). Collectively, these findings show that while canonical ASC-dependent inflammasome pathways do play some role in the cytokine responses associated with severe SARS-CoV-2 pathogenesis, they do not significantly impact disease outcome.

### The pyroptosis effector gasdermin D is not required for pro-inflammatory cytokine release during SARS-CoV-2 infection in mice

**Fig. 2:**
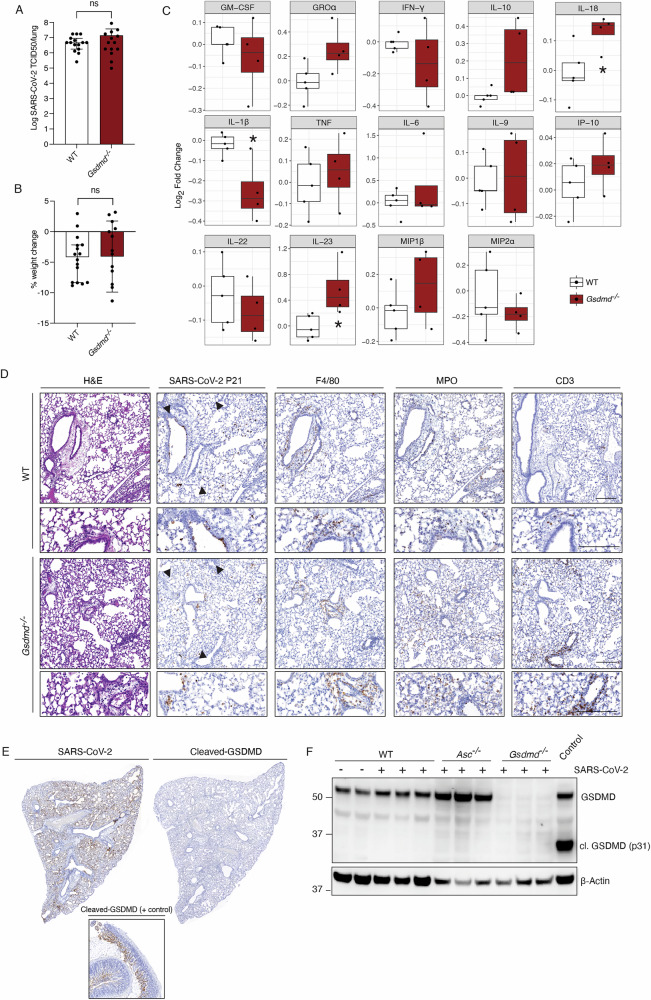
GSDMD is not essential to drive cytokine release and inflammation during SARS-CoV-2 infection. WT and *Gsdmd* knockout (*Gsdmd*^*−/−*^) mice were infected with 10^4^ TCID50/mouse of SARS-CoV-2 P21 and examined at 3 dpi for (**A**) lung viral burden by using a TCID50 assay, (**B**) percent weight change compared to initial weight (results are pooled from 2 independent experiments; *n* = 14–15 mice per genotype) and (**C**) levels of cytokines and chemokines measured by ELISA of lung homogenates (*n* = 4 mice per genotype). **D** WT and *Gsdmd*^*–/–*^ mice were challenged intranasally with 10^4^ TCID50/mouse of SARS-CoV-2 P21 and lungs were collected and fixed for histological analysis at 3 dpi. Representative images of haematoxylin and eosin (H&E) and immunohistochemistry (IHC) stained lungs testing for SARS-CoV-2 nucleocapsid, F4/80 (marker of macrophages), MPO (marker of neutrophils) and CD3 (marker of T cells) are shown. Images are representative of at least 3 animals per genotype. Black arrows point to exemplary SARS-CoV-2-positive cells. Scale bar = 250 µm. **E** 6-month-old WT mice were infected intranasally with 10^4^ TCID50/mouse of SARS-CoV-2 P21. At 3 dpi mice were euthanised and lungs were collected for histological analysis. Immunohistochemistry (IHC) was performed detecting SARS-CoV-2 nucleocapsid (left panel) or cleaved (i.e. activated) GSDMD (right panel). A positive control was performed for cleaved GSDMD using small intestine sections of mice infected with the enteric pathogen *Cryptosporidium* (bottom panel). **F** Western blot analysis of whole lungs from mock (–) or SARS-CoV-2 P21 infected (+) WT, *Asc*^*–/–*^ or *Gsdmd*^*–/–*^ mice at 3 days after intranasal SARS-CoV-2 P21 infection (10^4^ TCID50/mouse). Samples were probed for full-length and cleaved (i.e. activated) GSDMD. Probing for β-Actin is shown as a loading control. Unpaired two-tailed Student’s *t* test after log_10_ transformation (**A**), unpaired two-tailed Student’s *t* test (**B**), Wilcoxon rank-sum (**C**), statistical tests were performed (p* < 0.05).

Regardless of the upstream mechanisms responsible for the activation of pyroptosis and cytokine production, the cellular release of cytokines and cell lysis is thought to be primarily dependent on the activation of the pore forming protein GSDMD [43]. We found that GSDMD deficient animals (*Gsdmd*^*–/–*^) showed similar viral burden and weight loss compared to WT mice upon infection with SARS-CoV-2 P21 (Fig. 2A, B). Analysis of 25 cytokines and chemokines in lung homogenates from infected *Gsdmd*^*–/–*^ mice showed that the levels of IL-1β were slightly reduced compared to WT animals (Fig. 2C). Interestingly, however, IL-18 and IL-23 were increased in the lungs of these animals upon infection (Fig. 2C and S2A). These data reveal that GSDMD does not exert an essential role in the pathogenic pro-inflammatory cytokine release during SARS-CoV-2 infection in vivo.

We further compared lung histology of *Gsdmd*^*–/–*^ and WT mice at 3 dpi. Lung sections were stained with H&E and analysed by a board-certified pathologist. Three days post SARS-CoV-2 infection, both *Gsdmd*^*−/−*^ and WT mice displayed multifocal, acute alveolitis, multifocal pneumonia, as well as moderate to severe acute multifocal perivasculitis. IHC staining for nucleocapsid showed that SARS-CoV-2 P21 localised to the bronchiolar and alveolar epithelium in both WT and *Gsdmd*^*–/–*^ mice. MPO, CD3 and F4/80 staining revealed a similar pattern of myeloid cell and T cell infiltrates in WT and *Gsdmd*^*–/–*^ animals (Fig. 2D).

We next explored if severe SARS-CoV-2 disease was linked to cleavage (i.e. activation) of GSDMD in WT animals. In line with our previous results, no cleaved (i.e. activated) GSDMD was detected by IHC in WT animals with severe disease, which was confirmed by nucleocapsid staining (Fig. 2E). As a positive control, to ensure that our antibody detects cleaved GSDMD, we stained small intestine tissue from mice infected with the enteric pathogen *Cryptosporidium*, which is known to trigger pyroptosis and cause gasdermin D cleavage (activation) [44] (Fig. 2E bottom panel). To further investigate GSDMD cleavage during SARS-CoV-2 infection, we performed Western blot analysis of whole lung tissue extracts. Cleavage (i.e. activation) of GSDMD in the lungs of infected animals could not be detected in WT or *Asc*^*–/–*^ mice (Fig. 2F, original Western blots are presented in the supplemental information). Together with the findings from our genetic investigations, this indicates that the inflammation associated with severe SARS-CoV-2 disease does not lead to, or require, the activation of GSDMD in mice.

### Regulators of non-canonical pyroptosis are dispensable for SARS-CoV-2 infection-induced pathogenesis

**Fig. 3:**
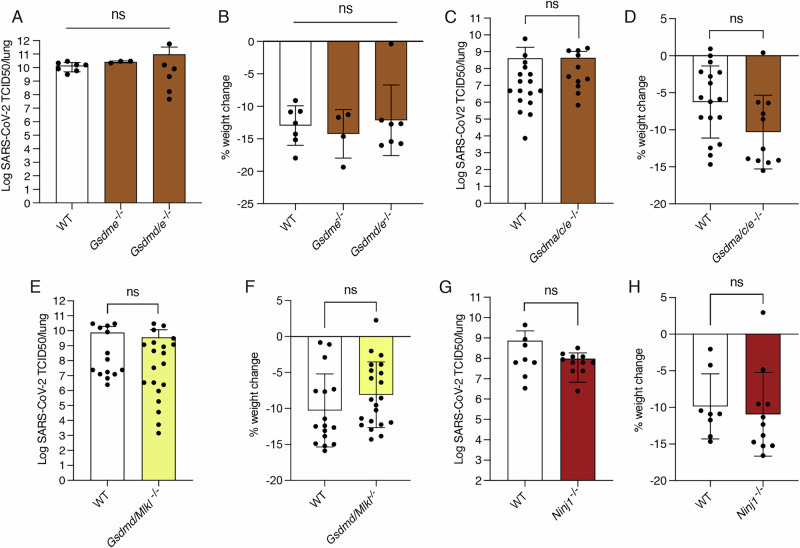
Inflammation driven by SARS-CoV-2 infection is independent of the non-canonical lytic forms of programmed cell death. WT*, Gsdme*^*–/–*^ *and Gsdmd/e*^*–/–*^ mice were infected intranasally with 10^4^ TCID50/mouse of SARS-CoV-2 P21 and examined at 3 dpi for (**A**) lung viral burden using a TCID50 assay and (**B**) percent weight change compared to initial weight (*n* = 4–7 per genotype). WT and *Gsdma/c/e*^*−/−*^ mice were challenged intranasally with 10^4^ TCID50/mouse of SARS-CoV-2 P21 and examined at 3 dpi for (**C**) lung viral burden using a TCID50 assay and (**D**) percent weight change compared to initial weight (*n* = 11–17 mice per genotype). WT and *Gsdmd/Mlkl* knockout (*Gsdmd/Mlkl*^*–/–*^) mice were infected with 10^4^ TCID50/mouse of SARS-CoV-2 P21 and examined at 3 dpi for (**E**) lung viral burden by using a TCID50 assay and (**F**) percent weight change compared to initial weight (*n* = 14–19 mice per genotype). WT and *Ninj1* knockout (*Ninj1*^*–/–*^) mice were infected with 10^4^ TCID50/mouse of SARS-CoV-2 P21 and examined at 3 dpi for (**G**) lung viral burden by using a TCID50 assay and (**H**) percent weight change compared to initial weight (*n* = 8–11 mice per genotype). Data are presented as mean ± SD. Statistical analyses were performed by using one-way ANOVA with multiple comparisons after log_10_ transformation (**A**), one-way ANOVA with multiple comparisons (**B**), unpaired two-tailed Student’s *t* test after log_10_ transformation (**C**, **E**, **G**) and unpaired two-tailed Student’s *t* test (**D**, **F**, **H**).

We further investigated the role of non-canonical pyroptosis pathways, including those involving GSDME activation. We infected *Gsdme*^*–/–*^ mice with SARS-CoV-2 but found no difference in disease phenotype compared to infected control WT animals (Fig. 3A–D). Furthermore, compound loss of GSDMD/E (*Gsdmd*^*–/–*^*/e*^*–/–*^, short *Gsdmd/e*^*–/–*^) and GSDMA/C/E (*Gsdma*^*–/–*^*/c*^*–/–*^*/e*^*–/–*^, short *Gsdma/c/e*^*–/–*^) did not alter disease phenotypes compared to SARS-CoV-2 infected WT mice (Fig. 3A–D).

Functional overlap of programmed cell death pathways, particularly in response to infection with certain pathogens, has emerged as a new paradigm in recent years [45, 46]. To examine if necroptosis may compensate for the loss of pyroptosis, we infected mice lacking essential effectors of both of these lytic programmed (regulated) cell death processes, namely GSDMD and MLKL, with SARS-CoV-2 P21. Compound mutant *Gsdmd*^*–/–*^*/Mlkl*^*–/–*^ (*Gsdmd/Mlkl*^*–/–*^) mice showed similar disease phenotypes compared to infected WT animals (Fig. 3E, F).

We next extended our investigation to examine mice that were deficient in NINJ1, a protein that is critical for plasma membrane rupture and release of high molecular weight DAMP during the final stages of pyroptosis, apoptosis, ferroptosis and accidental cell lysis, but not during necroptosis [47, 48]. Of note, *Ninj1*^*−/−*^ mice showed similar disease phenotypes upon SARS-CoV-2 infection as WT mice (Fig. 3G, H).

### Cytokine processing during SARS-CoV-2 infection occurs independently of caspases-1/-11/-12

**Fig. 4:**
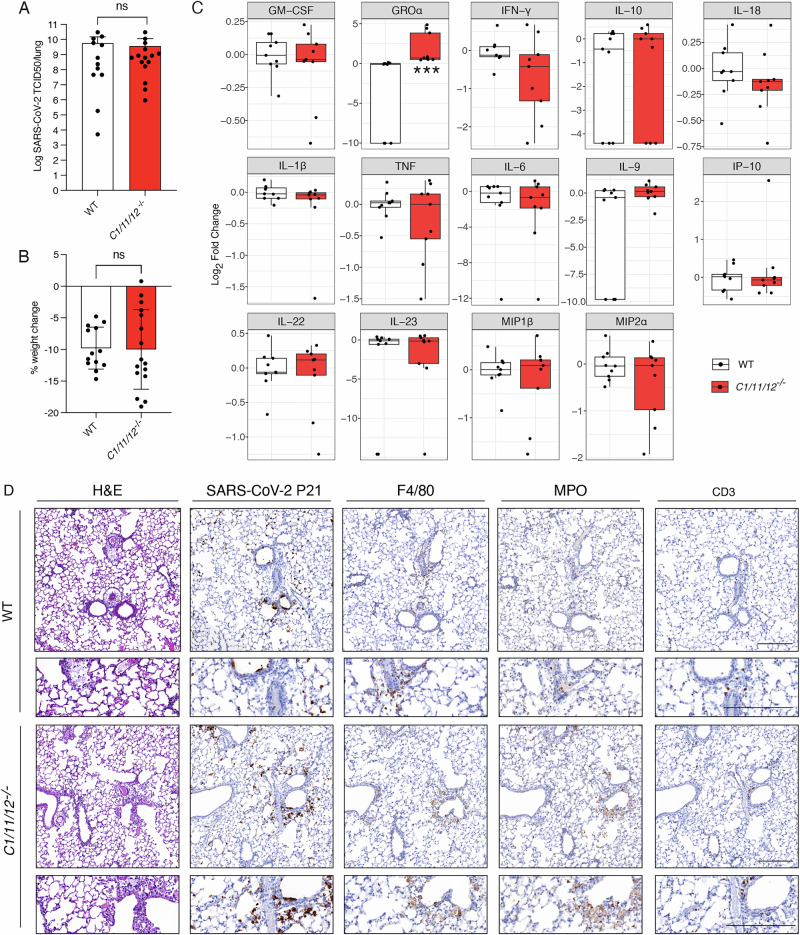
Caspases-1/-11/-12 do not contribute to SARS-CoV-2 driven disease. WT and *Caspase-1,-11,-12* triple knockout (*C1/11/12*^*–/–*^) mice were infected with 10^4^ TCID50/mouse of SARS-CoV-2 P21 and examined at 3 dpi for (**A**) lung viral burden by using a TCID50 assay, (**B**) percent weight change compared to initial weight (*n* = 12–15 mice per genotype), (**C**) levels of cytokines and chemokines were measured by ELISA of lung homogenates of WT and knockout animals at 3 dpi (*n* = 9 mice per genotype; data are presented as mean ± SD) and (**D**) histological analysis of fixed lungs. Representative images of haematoxylin and eosin (H&E) stained and immunohistochemistry (IHC) stained lungs testing for SARS-CoV-2 nucleocapsid, F4/80 (marker of macrophages), MPO (marker of neutrophils) and CD3 (marker of T cells) are shown. Images are representative of at least 3 animals per genotype. Black arrows point to exemplary SARS-CoV-2-positive cells. Scale bar = 250 µm. Statistical analyses were performed by unpaired two-tailed Student’s *t* test after log_10_ transformation (**A**), unpaired two-tailed Student’s *t* test (**B**), Wilcoxon rank-sum (**C**) (***p < 0.001).

Inflammasome and pyroptosis signalling have been implicated in the pathogenesis of COVID-19 because of their known link to the release of bio-active IL-1β and IL-18. Both of these cytokines have been associated with increased severity of disease [5, 49, 50]. The prevailing dogma is that these cytokines are obligatorily released downstream of inflammasome signalling and processed into their bioactive forms by caspases-1, -11 [51–53]. We therefore used gene-targeted mice lacking these caspases, as well as caspase-12 (*C1*^*–/–*^*/11*^*–/–*^*/12*^*–/–*^*, short C1/11/12*^*–/–*^*)*, to determine their overall contribution to severe SARS-CoV-2 disease in our mouse model. SARS-CoV-2 P21 infected *C1/11/12*^*−/−*^ mice showed similar viral burdens and weight loss compared to infected WT mice (Fig. 4A, B). To better understand the role of caspases-1, -11, and -12 during SARS-CoV-2 infection, we assessed viral burdens at 6 dpi. However, no significant differences were observed between *C1/11/12*^*−/−*^ versus WT mice, further indicating that the inflammatory caspases are not critical for viral load or clearance (Fig. S3A). Analysis of 25 cytokines and chemokines showed that upon SARS-CoV-2 infection, *C1/11/12*^*−/−*^ mice mounted a similar cytokine/chemokine response to infected WT controls, with only GROα being slightly elevated in the knockout mice (Fig. 4C, S3B). This finding indicates that processing and release of pro-inflammatory IL-1β and IL-18 during SARS-CoV-2 infection can occur independently of the three inflammatory caspases C1/-11/-12. Moreover, histological examination confirmed similar lung pathology and immune cell infiltrates in SARS-CoV-2 infected *C1/11/12*^*−/−*^ and WT mice (Fig. 4D). These results show that all components of the pyroptosis machinery, from ASC-dependent inflammasome sensors to catalytic pro-inflammatory caspases and the pore-forming effector proteins, are not essential to drive SARS-CoV-2 viremia or disease manifestations.

Similar to COVID-19 in humans, SARS-CoV-2 P21-driven disease in mice becomes more pronounced with increased age [30]. Upon infection with P21, the majority of WT animals (>10 weeks-old) lose more than 20% body weight, reaching ethical end-point by 4–6 dpi [30]. To further assess the role of canonical and non-canonical pyroptotic proteins in SARS-CoV-2-driven disease and viral clearance, we have infected aged (>6 months-old) *C1/11/12*^*−/−*^, *Gsdmd/e*^*–/–*^, *Il-18*^*–/–*^ and *Gsdmd/Mlkl*^*–/–*^ animals with P21. Compared to aged WT mice, none of the knockout animals showed significant differences in survival rates (Fig. S3C), indicating that the removal of canonical as well as non-canonical regulators and effectors of pyroptosis, even in combination with the loss of critical effectors of necroptosis, did not significantly affect age-dependent fatal SARS-CoV-2-driven disease.

### IL1-β but not IL-18 contributes to severe SARS-CoV-2-mediated disease

**Fig. 5:**
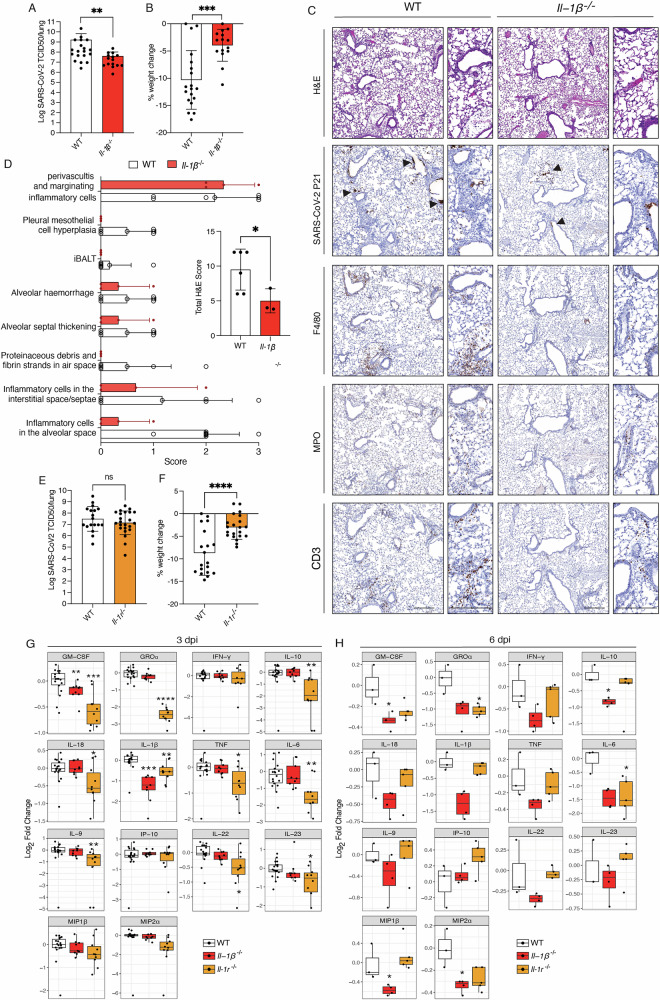
*Il-1*β knockout but not *Il-18* knockout mice are protected from severe SARS-CoV-2 disease and have reduced viral burden. WT and *Il-1β*^*−/−*^ mice were infected with 10^4^ TCID50/mouse of SARS-CoV-2 P21 and examined at 3 dpi for (**A**) lung viral burden by using a TCID50 assay, (**B**) percent weight change compared to initial weight (*n* = 15–19 mice per genotype) and (**C**) examination of lung pathology via histological assessment of fixed lungs. Representative images of haematoxylin and eosin (H&E) and immunohistochemistry (IHC) stained lungs testing for SARS-CoV-2 nucleocapsid, F4/80 (marker of macrophages), MPO (marker of neutrophils) and CD3 (marker of T cells). Histological images are representative of at least 3 animals per genotype. Black arrows point to exemplary SARS-CoV-2-positive cells. Scale bar = 250 µm. **D** Histological changes based on H&E staining from (**C**) were graded by an American board-certified pathologist who was blinded to the genotypes of the mice. The scores are based on the percentage of lesions: 0 = normal, 1 < 10%, 2 = 10–25%, 3 = 25–50%, 4 > 50%. Sum of the histological scores of WT and *Il-1β*^*–/–*^ mice are shown on the right. **E**–**G** WT and *Il-1r*^*–/–*^ mice were infected with 10^4^ TCID50/mouse of SARS-CoV-2 P21 and examined at 3 dpi for (**E**) lung viral burden by using a TCID50 assay, (**F**) percent weight change compared to initial weight (*n* = 11–16 mice per genotype) and (**G**) levels of cytokines and chemokines were measured by ELISA of lung homogenates (*n* = 8–18 mice per genotype). **H** The levels of cytokines and chemokines of lung homogenates were measured by ELISA of WT and knockout animals 6 days post SARS-CoV-2 P21 infection (*n* = 3–5 mice per genotype). Data are presented as mean ± SD. Statistical analyses were performed by unpaired two-tailed Student’s *t* test after log_10_ transformation (**A**, **E**), unpaired two-tailed Student’s *t* test (**B**, **F**), Wilcoxon rank-sum (**G**, **H**); In (**G**, **H**), significance is shown relative to WT mice (* < 0.05; **p < 0.01; ***p < 0.001).

Our results show that SARS-CoV-2 driven inflammation and cytokine release in vivo are independent of key components of the pyroptosis machinery. Several studies reported an association between IL-1β and IL-18 serum levels and severe COVID-19 [6, 7, 36]. Pyroptosis and caspases-1/-11/-12 are linked to IL-1β and IL-18 processing. So, if these inflammatory cytokines are critically involved in severe COVID-19, the results from our studies using gene targeted mice indicate a disconnect between the processing and release of IL-1β and IL-18 from inflammasomes, caspases-1/11/12 and pyroptosis. To confirm that one or both of these cytokines do indeed contribute to SARS-CoV-2 driven disease in our animal model, we infected IL-1β (*Il-1β*^*–/–*^) and IL-18 deficient (*Il-18*^*–/–*^) mice with SARS-CoV-2 P21. *Il-18*^*–/–*^ mice were not significantly protected from SARS-CoV-2 infection, displaying similar viral burden and disease compared to P21 infected WT animals (Fig. S4A–C). In contrast, *Il-1β*^*–/–*^ mice had significantly lower viral burden in the lungs and exhibited less weight loss compared to P21 infected WT animals (Fig. 5A, B).

To understand the cellular host responses linked to better outcomes in SARS-CoV-2 infected *Il-1β*^*–/–*^ mice, we examined lung histology at 3 dpi. Lung sections were stained with H&E and analysed by a board-certified pathologist. Three days post SARS-CoV-2 P21 infection, WT mice had mild to severe acute multifocal perivasculitis, interstitial pneumonia and necrotizing alveolitis. Infected IL-1β deficient mice also showed perivasculitis, moderate interstitial pneumonia, but no alveolitis (Fig. 5C, D). IHC staining for SARS-CoV-2 nucleocapsid showed that P21 virus localised to the bronchiolar and alveolar epithelium and macrophages in both WT and *Il-1β*^*–/–*^ mice. Histological scoring showed that in contrast to WT controls, P21 infected *Il-1β*^*−/−*^ mice did not present with signs of pleural mesothelial hyperplasia, iBALT (inducible Bronchus-Associated Lymphoid Tissues) or proteinaceous debris in the air space. These differences amounted to an overall significantly reduced histological score of disease (Fig. 5C, D). These findings corroborate the human correlative data and prove for the first time, using gene knockout mice, that IL-1β plays a critical role during severe SARS-CoV-2-induced disease.

Inflammatory IL-1 signalling is activated through binding of IL-1 family cytokines to the membrane bound, type I interleukin-1 receptor (IL-1R) [54]. Interestingly, while *Il-1r*^*–/–*^ animals were also protected from severe SARS-CoV-2-induced disease, as revealed by a reduction in weight loss, these animals did not have decreased viral burden but were similar to SARS-CoV-2 P21 infected WT mice in this respect (Fig. 5E, F).

To identify the effects of the absence of critical components of the IL-1 signalling pathway on the pro-inflammatory cytokine response, we quantified the levels of 25 cytokines and chemokines in the lungs of WT, *Il-1β*^*–/–*^ and *Il-1r*^*–/–*^ animals at 3 dpi with SARS-CoV-2 P21. The absence of IL-1β led to reductions in GM-CSF, IL17a and IL-5. *Il-1r*^*−/−*^ animals tended to express lower levels of a wider range of cytokines and chemokines compared to the IL-1β deficient mice (Fig. 5G, S5A).

To better understand the differences observed between *Il-1β*^*–/–*^ and *Il-1r*^*–/–*^ mice, we extended our analysis to multiple time points after SARS-CoV-2 P21 infection. At 24 h post-infection the two sets of knockout mice showed comparable viral burden to those observed in WT mice (Fig. S5B). While in WT mice most cytokines measured by ELISA were increased 24 h post SARS-CoV-2 P21 infection compared to mock WT animals (Fig. S5C), *Il-1β*^*–/–*^ and *Il-1r*^*–/–*^ mice only differed in levels of IL-22 at this early time point (Fig. S5D). However, while viral loads at 6 dpi were similar between *Il-1β*^*–/–*^ and *Il-1r*^*–/–*^ animals (Fig. S5E), the reduction in cytokines was preserved, continuing for 6 days post-infection with SARS-CoV-2 P21, and was more profound at later time points in IL-1β deficient mice compared to *Il-1r*^*–/–*^ and WT animals (Fig. 5H, S5F).

### Age-related severity of SARS-CoV-2 disease can be reduced by the absence of IL-1β

**Fig. 6:**
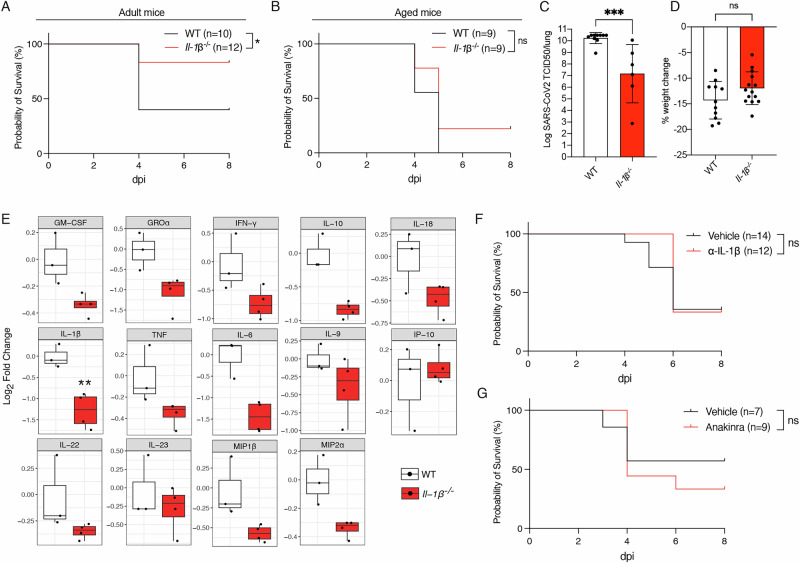
Age-associated SARS-CoV-2-induced disease is reduced in *Il-1β*^*–/–*^*mice*. **A** WT and *Il-1β*^*–/–*^ adult mice (adult = 11–12 weeks) were intranasally infected with 10^4^ TCID50/mouse SARS-CoV-2 P21 and monitored to determine the proportion of mice that became severely ill, reaching predetermined ethical endpoint (*n* = 10–12 mice per genotype). Aged ( > 6 month-old) WT and *Il-1β*^*–/–*^ mice were infected with 10^4^ TCID50/mouse of SARS-CoV-2 P21 and monitored for (**B**) proportion of mice that became severely ill, reaching predetermined ethical endpoint (*n* = 9 mice per genotype), (**C**) lung viral burden was examined at 3 dpi by using a TCID50 assay, (**D**) percent weight change at 3 dpi compared to initial weight (*n* = 6–10 mice per genotype) and (**E**) the levels of cytokines and chemokines were measured by ELISA of lung homogenates of WT and knockout animals 6 days post SARS-CoV-2 P21 infection (*n* = 3–4 mice per genotype). **F** WT and *Il-1β*^*–/–*^ adult mice (adult = 11–12 weeks) were treated with anti-Il-1β monoclonal antibodies and intranasally infected with 10^4^ TCID50/mouse SARS-CoV-2 P21 and monitored to determine the proportion of mice that became severely ill, reaching predetermined ethical endpoint (*n* = 14–12 mice per group). **G** WT and *Il-1β*^*–/–*^ adult mice (adult = 11–12 weeks) were treated with anakinra and intranasally infected with 10^4^ TCID50/mouse SARS-CoV-2 P21 and monitored to determine the proportion of mice that became severely ill, reaching predetermined ethical endpoint (*n* = 7–9 mice per group). Data are presented as mean ± SD. Statistical analyses were performed by Log-rank Mantel–Cox test (**A**, **B**, **F**, **G**), unpaired Mann–Whitney test after log_10_ transformation (**C**), unpaired two-tailed Student’s *t* test (**D**) and Wilcoxon rank-sum (**E**). In (**E**), significance is shown relative to WT mice (**p < 0.01).

To test whether the absence of IL-1β could protect against age-associated severity of SARS-CoV-2 driven disease, we infected 11 week-old *Il-1β*^*–/–*^ and control WT mice with P21. IL-1β deficient mice were more likely to survive this infection compared to WT controls (Fig. 6A), however, this survival advantage was attenuated in 6-month-old mice (Fig. 6B). Despite this, 6-month-old IL-1β deficient mice showed significantly lower viral burden in the lungs at the peak of infection (3 dpi) compared to WT controls (Fig. 6C, D). Interestingly, in animals older than 6 months, while multiple cytokines were decreased at 3 dpi, the only cytokine found to be significantly reduced in lung homogenates from *Il-1β*^*–/–*^ mice was IL-1β itself (Fig. 6E, Fig. S6A). To better understand the role of IL-1β in age-associated SARS-CoV-2 pathology, we compared the levels of this cytokine in lung homogenates between infected young versus aged WT animals. Notably, aged animals did not exhibit elevated IL-1β levels in the lungs following SARS-CoV-2 P21 infection compared to their younger counterparts (Fig. S6B). These results indicate that while IL-1β plays a critical role in disease severity, knockout of this cytokine in aged animals is not sufficient on its own to ameliorate age-associated COVID-19 pathology.

### Pharmacological inhibition of IL-1 signalling is not sufficient to prevent SARS-CoV-2-induced mortality

To assess the efficacy of anti-IL-1β therapeutics in our severe COVID-19 mouse model, we administered a single dose of IL-1β-neutralising monoclonal antibodies, analogous to the clinically used human antibody Canakinumab, to 10- to 12-week-old WT mice on the day of infection with P21. Such inhibition of IL-1β alone was not sufficient to prevent mortality in P21 infected animals although a trend towards reduced mortality was observed (Fig. 6F).

To better understand if IL-1 receptor antagonism could provide greater protection against SARS-CoV-2-driven disease, we treated P21 infected WT mice daily from the day of infection with 10 mg/kg anakinra, a dose that is pharmacologically active in mice and reflects therapeutic levels used in humans [55, 56]. Akin to treatment with anti-IL-1β, anakinra did not improve survival after SARS-CoV-2 P21 infection in our model (Fig. 6G). Thus, more profound inhibition of IL-1β and/or concomitant inhibition of other pathogenic cytokines should be explored to combat severe COVID-19.

## Discussion

COVID-19 morbidity and mortality are driven by dysregulated host cytokine signalling. The pro-inflammatory cytokines IL-18 and IL-1β have been implicated in disease pathogenesis. Based on in vitro experiments, or mouse models with non-physiological overexpression of hACE2, it has been proposed that NLRP3 inflammasome activation and pyroptosis are responsible for the exaggerated production of IL-18 and IL-1β [5, 9, 10, 49, 57]. We confirmed in our mouse model that IL-1 signalling contributes to severe SARS-CoV-2 disease through IL-1β, whereas IL-18 plays a negligible role. Furthermore, our data challenge the notion that NLRP3, NLRP1 and other ASC-dependent inflammasomes are essential for IL-1β-driven SARS-CoV-2 disease pathogenesis. Although deletion of ASC and GSDMD slightly reduced the levels of IL-1β in the lungs of SARS-CoV-2 P21 infected mice compared to WT controls, this reduction was quantitatively minor and not sufficient to improve disease outcome. These findings align with the literature, confirming that GSDMD and ASC lead to IL-1β release during SARS-CoV-2 infection. However, our study extends this understanding by utilizing genetic murine models to demonstrate that the reduction in IL-1β that is dependent on the inflammasome is insufficient to mitigate disease severity in vivo. This distinction underscores the importance of our work, highlighting how disease relevance can be misrepresented in in vitro-only models. Collectively, our study provides genetic in vivo evidence, utilizing a model based on a clinical SARS-CoV-2 isolate and physiological ACE2 expression levels, demonstrating that inflammasomes and GSDMD are not required for achieving pathogenic lung IL-1β levels. Furthermore, we show that neither inflammasomes nor GSDMD are essential for disease pathogenesis or viral dissemination in vivo. Interestingly, in severe influenza A virus (IAV) infection, GSDMD promotes disease in mice, but infection-induced release of IL-1β is also independent of GSDMD [58].

We also found that effectors of pyroptosis, including GSDMD, GSDME and GSDMA/C/E, are not required to induce severe SARS-CoV-2 disease in our model. As we have previously shown, necroptosis was also dispensable for causing severe disease [29] and even combined deletion of genes for the essential effectors for pyroptosis and necroptosis (*Gsdmd/Mlkl*^*−/−*^) did not diminish pathology. We further demonstrated that lytic programmed cell death overall does not play a prominent role in SARS-CoV-2 driven disease using mice lacking NINJ1, which is essential for plasma membrane rupture in pyroptosis, ferroptosis and late-stage apoptosis [47]. Importantly, caspases-1, -11 and -12 were not required for the release of IL-1β during severe SARS-CoV-2 disease. Collectively, these findings indicate that processes independent of lytic programmed cell death must be able to facilitate IL-1β processing and release during SARS-CoV-2 infection. Several potential mechanisms have been identified (reviewed in [59]), suggesting that this cytokine can be converted into its bio-active forms downstream of pathways other than canonical inflammasome and gasdermin-effected pyroptosis (or other forms of regulated lytic cell death). Recent studies have broadened our understanding of the caspases capable of proteolytically activate IL-1β and regulate cytokine transcription [60], making it tempting to speculate that proteolytic processing of IL-1β/α during SARS-CoV-2 infection might be mediated by caspases other than caspases-1/11/12.

Although studies have suggested that NLPR3 activation and GSDMD serve as predictive markers for severity of COVID-19 [5, 49], these studies were solely conducted in vitro. Published in vivo studies using *Nlrp3*^*−/−*^ animals, utilised adeno-associated virus (AAV) to deliver hACE2 prior to SARS-CoV-2 infection [57], an additional process that adds complexities, such as triggering inflammatory pathways on its own. To date, clinical trials testing NLRP3 inhibitors in COVID-19 patients have been inconclusive, only showing subtle changes in mortality and not being successful in improving APACHE II scores compared to treatment with standard-of-care alone [61]. Our data complement this work indicating that inflammasomes and pyroptosis are not promising targets for the treatment of severe COVID-19.

Given the critical role of IL-1β in severe disease in our SARS-CoV-2 infection model we compared the effects of IL-1β deficiency to the loss of its cognate receptor IL-1R during SARS-CoV-2 infection. Although there were similarities in disease amelioration compared to infected WT mice, loss of IL-1β caused a more profound drop in inflammatory cytokine levels at later time points during infection compared to the absence of its cognate receptor. This indicates that complete ablation of all IL-1R binding cytokines is not more beneficial than IL-1β deletion alone. This conclusion is further supported by the lack of a significant reduction in viral burden in IL-1R deficient mice compared to WT controls. IL-1R signalling can be activated by both IL-1α and IL-1β, and even though both cytokines trigger the same receptor, they are known to be non-redundant in vivo [62]. Discrepancies between IL-1R and IL-1β deficiency phenotypes are well documented in the literature and this translates to clinical differences between IL-1R blocking and IL-1 blocking antibodies in the management of disease [63]. Interestingly, other inflammatory cytokines, including TNF, IL-6, and IFN-γ, are also reduced in *Il-1β*^*−/−*^ animals, potentially contributing to the observed reduction in disease severity. These findings align with numerous studies highlighting the role of IL-1β in driving the production of pro-inflammatory cytokines across diverse contexts [54, 64–66]. Notably, our previous work has shown that *Tnf*^*−/−*^ animals are similarly protected from severe disease in our SARS-CoV-2 model [30]. This indicates that simultaneous reduction of TNF that acts complementarily to IL-1β, may also be a factor leading to improved disease outcomes in *Il-1β*^*−/−*^ mice.

Several studies both in mice and human patients, have evaluated the therapeutic potential of inhibiting IL-1 signalling during SARS-CoV-2 infection using anakinra, an IL-1 receptor antagonist (IL-1RA). In preclinical models utilising K18-hACE2 transgenic mice, treatment with IL-1RA has been associated with improved survival [67]. However, this model elicits severe disease through neuroinvasiveness, systemic viral dissemination [68], and encephalitis [69], which differs significantly from the predominant clinical manifestations of COVID-19 in humans. In K18-hACE2 mice, NLRP3 inflammasome activation and IL-1β release are observed late in infection (7 dpi), coinciding with fatal disease progression [67]. Paradoxically, treatment with IL-1RA in this model resulted in increased viral loads and elevated levels of NLRP3 and cleaved caspase-1, suggesting potential activation of inflammatory pathways despite therapeutic intervention [67]. Clinical trials have also examined the therapeutic impact of inhibiting IL-1 signalling during severe COVID-19, with conflicting results [70–74]. These discrepancies may reflect differences in patient cohorts, disease severity, or timing of intervention, underscoring the complexity of targeting IL-1-mediated inflammation in the context of SARS-CoV-2 infection.

Our data, derived from comparing IL-1β knockout mice with IL-1R knockout mice, revealed that removal of IL-1β alone leads to a more prominent reduction of pro-inflammatory cytokines than loss of IL-1R. We further assessed the impact of both IL-1β neutralising monoclonal antibodies and anakinra in our model. We could not recapitulate the survival advantage conferred by genetic loss of IL-1β using a validated IL-1β neutralising antibody, and IL-1R also had no impact on severity of disease. Several factors could contribute to this discrepancy, most notably inadequate neutralisation of IL-1β and IL-1R within the relevant tissue(s) by using these agents. Another therapeutic avenue would be to target the upstream mechanisms responsible for IL-1β production. Here, we have ruled out some candidate pathways, including various forms of lytic programmed cell death, such as pyroptosis and necroptosis.

Given the importance of IL-1β in promoting severe SARS-CoV-2-induced disease in our model and mounting evidence for a critical role of this cytokine in driving severe human COVID-19 [36, 75–81], we further investigated the contribution of IL-1β in age-associated severe COVID-19. We have previously shown that genetic deletion of TNF diminished lethality in aged animals infected with P21, albeit only partially. This indicates that TNF is not solely responsible for severe age-associated SARS-CoV-2-driven disease. Here, we show that IL-1β is another key cytokine that contributes significantly to severe SARS-CoV-2-induced disease. The deletion of the *Il-1β* gene prevented mortality in young adult animals and attenuated the severity of disease in aged mice (>6 months). Why IL-1β deletion does not protect aged animals from mortality remains speculative and warrants further study. A recent study providing an atlas of the aging lung demonstrates that although *Il-1β* is upregulated in aged animals at the transcriptomic level, both *Il1r1* and its co-receptor (il1rap) are significantly downregulated. Conversely, *Il1r2*, a decoy receptor operating within the IL-1 signalling pathway, is upregulated [82]. This suggests a potential attenuation in the signalling response to IL1β in the lungs of aged mice.

In conclusion, we have shown a critical pathogenic role for IL-1β during severe SARS-CoV-2 disease in our mouse model. We provide evidence that pyroptotic cell death is not a major driver responsible for IL-1β production. This underscores the importance of further work to determine what process(es) promote(s) IL-1β release to fuel the cytokine storm driven disease in SARS-CoV-2 infected mice. Identification of the critical upstream signalling pathways may identify novel therapeutic targets that could underpin novel therapeutic strategies to abrogate IL-1β production and thus overcome potential issues with inadequate blockade using neutralizing antibodies. Finally, data available overall may suggest that combined neutralisation of IL-1β and TNF may be superior in preventing severe SARS-CoV-2-induced pathology compared to inhibiting only one of these cytokines.

## Materials and methods

### Mice

All animal strains and experiments were reviewed and approved by the Walter and Eliza Hall Institute of Medical Research Animal Ethics Committee. Male or female WT and gene-targeted mice were bred and maintained on a C57BL/6 J background in the Specific Pathogen Free (SPF) Physical Containment Level 2 (PC2) Bioresources Facility at The Walter and Eliza Hall Institute of Medical Research (WEHI). WT mice were always co-housed in the same breeding rooms as the gene-targeted mice. Young (6–8 week-old), adult (10–12 week-old) and aged (>6 month-old) were used.

All procedures involving animals and live SARS-CoV-2 strains were conducted in an OGTR-approved Physical Containment Level 3 (PC3) facility at WEHI (Cert-3621). Mice were transferred to the PC3 laboratory for all SARS-CoV-2 infection experiments at least 4 days prior to the start of experiments. Animals were age- and sex-matched within experiments (both sexes were used). Experimental mice were housed in individually ventilated microisolator cages under level 3 biological containment conditions with a 12-hour light/dark cycle. Mice were provided with WEHI mouse breeder cubes (Ridley Agri Products) and sterile acidified water *ad libitum*.

The *Ninj1*^*–/–*^ mice were generated by the Melbourne Advanced Gene Editing Centre (MAGEC) laboratory (WEHI) on a C57BL/6 J background. Specifically, to generate *Ninj1*^*−/−*^ mice, 20 ng/mL of *Cas9* mRNA, 10 ng/mL of sgRNAs (CACACACTGGTCTCTAGCGG and TGTCGACAGCTGGAGTAATA) were injected into the pronucleus of fertilised one-cell stage embryos generated from wildtype C57BL6/J breeders. After 24 h, two-cell stage embryos were transferred into the uteri of pseudo-pregnant female mice. Viable offspring were genotyped by next-generation sequencing.

### SARS-CoV-2 strains

The SARS-CoV-2 VIC2089 clinical isolate (hCoV-19/Australia/VIC2089/2020) was obtained from the Victorian Infectious Disease Reference Laboratory (VIDLR). Viral passages were achieved by serial passage of VIC2089 through successive cohorts of 6–8 week-old C57BL/6 J (WT) mice. Briefly, mice were infected with the SARS-CoV-2 clinical isolate intranasally. At 3 dpi, mice were euthanised, and lungs harvested and homogenised in a bullet blender (Next Advance Inc) in 1 mL Dulbecco’s modified Eagle’s medium (DMEM) (Gibco/ThermoFisher) containing steel homogenisation beads (Next Advance Inc). Samples were clarified by centrifugation at 10,000 × *g* for 5 min before intranasal delivery of 30 µL lung homogenate into a new cohort of naïve C57BL/6 J mice. This process was repeated a further 20 times to obtain the SARS-CoV-2 VIC2089 P21 isolate. Lung homogenates from all passages were stored at –80 °C.

### Infection of mice with SARS-CoV-2 P21

In total, 6–8 week-old, or 6 month-old mice were anesthetised with methoxyflurane and inoculated intranasally with 30 μL SARS-CoV-2 P21 suspension. Virus stocks were diluted in serum free DMEM to a final concentration of 10^4^ TCID50/mouse. After infection, animals were visually checked and weighed daily for a minimum of 10 days. Mice were euthanised at the indicated times post-infection by CO_2_ asphyxiation. For histological analysis, animals were euthanised by cervical dislocation. Lungs were collected and stored at -80°C in serum-free DMEM until further processing.

### Measurement of viral load by determining 50% tissue culture infectious dose (TCID_50_)

TCID_50_ was performed as previously described in [83]. Briefly, Vero African green monkey kidney epithelial cells, purchased from ATCC (clone CCL-81), were seeded in flat bottom 96-well plates (1.75 × 10^4^ cells/well) and left to adhere overnight at 37 °C/5% CO_2_. Cells were washed twice with PBS and transferred into serum-free DMEM containing TPCK trypsin (0.5 µg/mL working concentration). Infected organs were defrosted, homogenised and then clarified by centrifugation at 10,000 × *g* for 5 min at 4 °C. Supernatant was added to the first row of cells at a ratio of 1:7, followed by 9 rounds of 1:7 serial dilutions in the following rows. Cells were incubated at 37°C/5% CO_2_ for 4 days until virus-induced cytopathic effects (CPE) could be scored. For scoring of CPE, researchers were blinded to the experimental groups. TCID_50_ was calculated using the Spearman & Kärber algorithm as described in [83]. All cell lines tested negative for mycoplasma contamination by PCR.

Animals that did not show productive infection (TCID50 under the limit of detection) were excluded in experiments that compared viral loads (TCID50) and weight loss between different viral strains or mouse genotypes.

### Histological analysis and immunohistochemical staining

Organs were harvested and fixed in 4% paraformaldehyde (PFA) for 24 h, followed by dehydration in 70% ethanol, paraffin embedding and sectioning. Slides were stained with either hematoxylin and eosin (H&E), or immunohistochemically stained with antibodies against CD3 (1:500, Agilent A045201), MPO (1:1000, Agilent A039829), F4/80 (1:1000, WEHI in-house antibody), or an antibody against SARS-CoV-2 nucleocapsid (1:4000, abcam ab271180) using the automated Omnis EnVision G2 template (Dako, Glostrup, Denmark). De-waxing was performed with Clearify Clearing Agent (Dako) and antigen retrieval was performed using EnVision FLEX TRS, High pH (Dako) at 97 °C for 30 min. Primary antibodies were diluted in EnVision Flex Antibody Diluent (Dako) and incubated at 32 °C for 60 min. HRP-labelled secondary antibodies (Invitrogen, Waltham, USA) were applied at 32 °C for 30 min. Slides were counter-stained with Mayer haematoxylin, dehydrated, cleared, and mounted with MM24 mounting medium (Surgipath-Leica, Buffalo Grove, IL, USA). Slides were scanned with an Aperio ScanScope AT slide scanner (Leica Microsystems, Wetzlar, Germany) and analysed by an American board-certified pathologist (Smitha Rose Georgy), who was blinded to the experimental groups, to describe histological changes. The scores are based on the percentage of lesions found in the lungs: 0 = normal, 1 < 10%, 2 = 10–25%, 3 = 25–50%, 4 > 50%.

### Lung cytokine and chemokine analysis

Lungs were thawed, homogenised and clarified by centrifugation at 10,000 × *g* for 5 min at 4 °C. Supernatants were pre-treated for 20 min with 1% Triton-X-100 for viral de-activation and the Cytokine & Chemokine 26-Plex Mouse ProcartaPlex Panel 1 (EPX260-26088-901) was used according to the manufacturer’s instructions. Briefly, 25 µL of clarified lung samples were diluted with 25 µL universal assay buffer, incubated with magnetic capture beads, washed, incubated with detection antibodies and SA-PE. Cytokines were recorded on a Bio-plex 200 system (Bio-Rad) and quantitated by comparison to a standard curve. Analysis was performed using R Studio.

### Western blot analysis

Total cell protein was isolated from whole mouse lungs using cell lysis buffer containing 20 mM Tris-HCl, pH 7.5, 135 mM NaCl, 1.5 mM Mg_2_Cl, 1 mM EGTA, 1% Triton X-100 (Sigma-Aldrich), 10% glycerol, EDTA-free protease inhibitor tablets (Roche, Basel, Switzerland), and phosphatase inhibitor tablets (Roche). Absolute protein content of clarified lysates was determined by using the BCA Protein Assay (Pierce), and equal quantities (10–30 μg) of total protein were separated under denaturing and reducing conditions (with 5% β-mercapto-ethanol) using 4–12% SDS-PAGE gels (Life Technologies). Proteins were transferred onto a PVDF membrane. These membranes were blocked with 5% skim milk (Devondale, Brunswick, Australia) in TBS with 0.05% Tween-20 (TBST) for 30 min, and proteins of interest were detected using the following primary antibodies: rabbit anti-GSDMD (Abcam, ab209845) and rabbit anti-beta-Actin (Cell Signalling, 13E5; used as a loading control). HRP-conjugated secondary goat anti-rabbit IgG antibodies (Southern Biotech, Birmingham, AL, USA) were then applied to membranes, which were subsequently washed in PBS with 0.05% Tween-20 (PBST) and then incubated with Immobilon Western Chemiluminescent HRP Substrate (Merck Millipore) and imaged using a ChemiDoc Touch Imaging System (Bio-Rad).

A positive control for GSMD activation was prepared by infecting bone marrow derived macrophages with Salmonella (SL1344) at MOI of 50, as previously described [45].

### Treatment of mice

For neutralisation of IL-1β, 10–12 week-old animals were treated intraperitoneally with 200 μg of monoclonal antibody (Bio X Cell BE0246) on the day of SARS-CoV-2 P21 infection. Control mice received 200 μg of Ig isotype matched control antibodies (Bio X Cell, BE0091). Animals were monitored daily for weight change and were euthanised upon reaching the humane endpoint (>20% weight loss).

Anakinra treatment was carried out as previously described [84]. Briefly, animals received daily intraperitoneal injections of anakinra (10 mg/kg) or vehicle (PBS), starting on the day of infection with SARS-CoV-2 P21.

### Quantification of data and statistical analyses

The assumption of normality for data distribution was evaluated using the Shapiro-Wilk test. For data that followed a normal distribution, an unpaired two-tailed Student’s *t* test was conducted to assess the significance of differences between two groups. When comparing multiple groups with one variable, one-way ANOVA followed by Tukey’s post hoc test was used to identify statistically significant differences. For data that did not follow a normal distribution, the Kruskal-Wallis test was applied for multiple group comparisons, followed by Dunn’s multiple comparisons test where applicable. For comparisons between two groups with non-normally distributed data, the Mann–Whitney U test was performed. Statistical analysis of cytokine data consisted of the Wilcoxon rank-sum test between group medians, with Bonferroni adjustment for multiple comparisons. Boxplots in figures depict the median and interquartile ranges.

Sample sizes (n) replicate numbers, and significance can be found in the figures and figure legends. For all statistical significance indications: *P < 0.05; **P < 0.01; ***P < 0.001; ****P < 0.0001; and ns, not statistically significant (*P* > 0.05).

For in vivo experiments, in accordance with the 3Rs, the smallest sample size was chosen that could give a significant difference (less than 0.05 type 1 error probability at 0.8 power). Pilot experiments were used to estimate the sample size such that an appropriate statistical test could yield significant results. The exact n numbers used in each experiment are indicated in the figure legends.

## Supplementary information

Supplementary Material

Original Data Files

## Data Availability

• Viral strains used in this study are available from the authors upon signing of a Materials Transfer Agreement (MTA). • All data generated or analysed during this study are included in this published article [and its supplementary information files]. • Full histological images reported in this paper will be shared by the lead contact upon request.
