# Supplementary material for: IL-1β drives SARS-CoV-2-induced disease independently of the inflammasome and pyroptosis signalling
Source: Cell Death Differ. 2025 Feb 28;32(7):1353–66. doi: 10.1038/s41418-025-01459-x (PMC12284219; doi:10.1038/s41418-025-01459-x)
Supplement: Supplementary file 1 — Supplementary Material [file 41418_2025_1459_MOESM1_ESM.pdf]

Supplementary Material

IL-1β drives SARS-CoV-2-induced disease independently of the inflammasome and pyroptosis signalling

Stefanie M. Bader[1,2], Lena Scherer[1], Jan Schaefer[1,2], James P. Cooney[1,2], Liana Mackiewicz[1], Merle Dayton[1], Smitha Rose Georgy[3], Kathryn C. Davidson[1,2], Cody C. Allison[1], Marco J. Herold[1,2,4,5], Andreas Strasser[1,2], Marc Pellegrini[1,2,6†*], Marcel Doerflinger[1,2†*]

Affiliations

[1] The Walter and Eliza Hall Institute of Medical Research (WEHI), Parkville, VIC 3052, Australia.

[2] Department of Medical Biology, University of Melbourne, Melbourne, Australia.

[3] Anatomic Pathology – Veterinary Biosciences, Melbourne Veterinary School, University of Melbourne, Werribee-3030 Australia

[4] Olivia Newton-John Cancer Research Institute, Heidelberg, Victoria, Australia

[5] School of Cancer Medicine, La Trobe University, Heidelberg, Victoria, Australia

[6] Centenary Institute and University of Technology Sydney, Faculty of Science, School of Life Sciences, Sydney, NSW, Australia.

† These authors contributed equally to this work

* To whom correspondence should be addressed:

Marcel Doerflinger, doerflinger.m@wehi.edu.au; Marc Pellegrini, m.pellegrini@centenary.org.au.

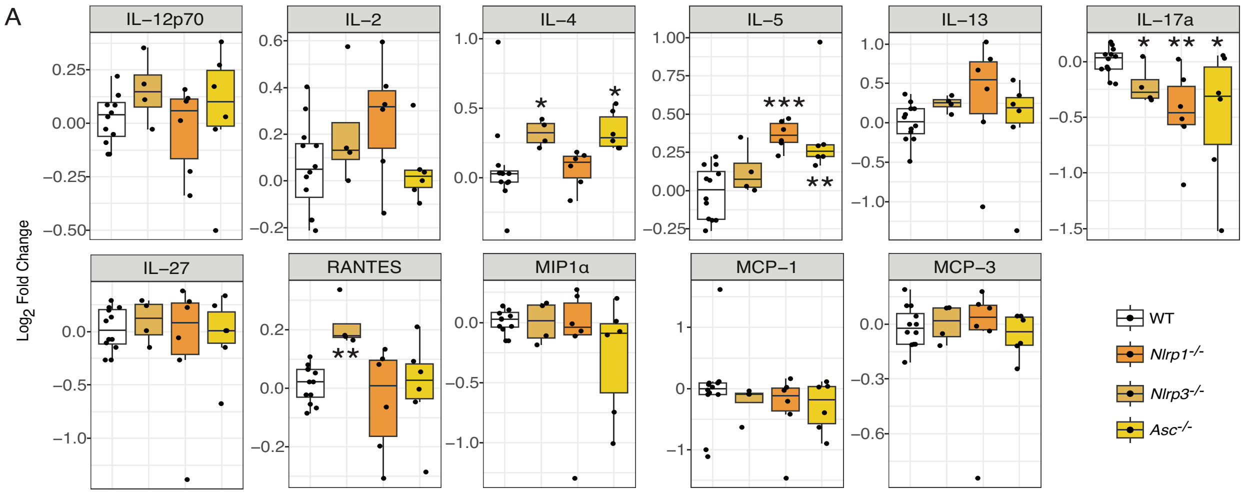
A
IL–12p70
IL–2
IL–4
IL–5
IL–13
IL–17a
IL–27
RANTES
MIP1α
MCP–1
MCP–3
Log2 Fold Change
WT
Nlrp1-/-
Nlrp3-/-
Asc-/-

-

Fig. S1: Severe inflammation driven by SARS-CoV-2 infection is independent of inflammasomes. (A) WT, *Nlrp3* (*Nlrp3*$^{-/-}$), *Nlrp1* (*Nlrp1*$^{-/-}$) and *Asc* (Asc$^{-/-}$) knockout mice were infected with 10^4 TCID50 of SARS-CoV-2 P21 and examined at 3 days post-infection (dpi) for levels of cytokines and chemokines, measured by ELISA of lung homogenates (n=5-10 mice per genotype). Wilcoxon rank-sum statistical tests were performed. Significance is shown relative to WT mice (* < 0.05; **p < 0.01).

A

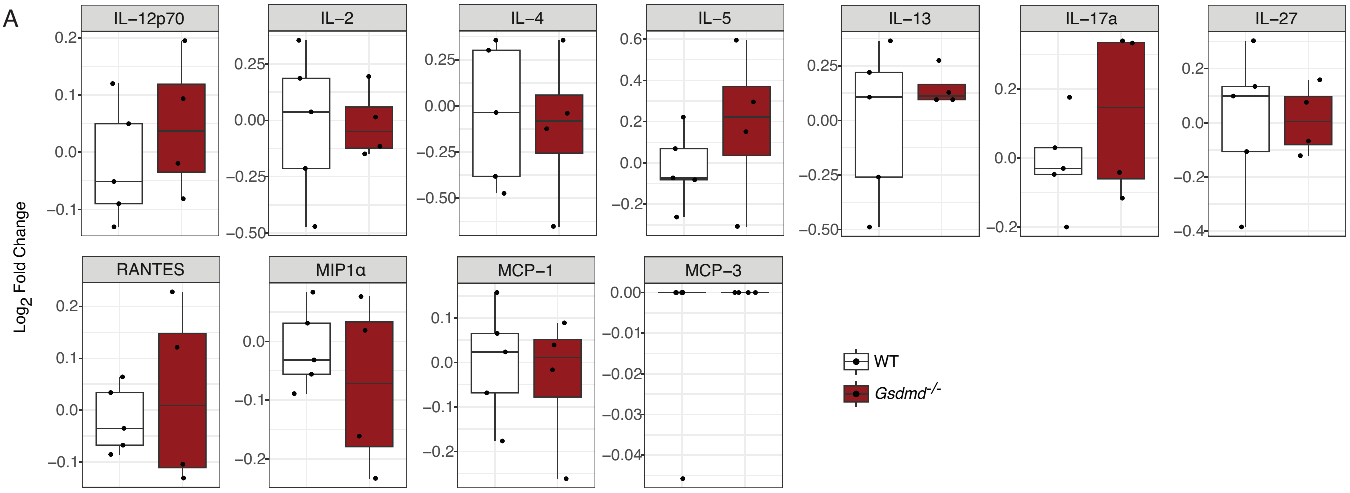

IL−12p70
IL−2
IL−4
IL−5
IL−13
IL−17a
IL−27
RANTES
MIP1α
MCP−1
MCP−3
Log2 Fold Change
WT
Gsdmd-/-

Fig. S2: GSDMD is not essential to drive cytokine release and inflammation during SARS-CoV-2 infection. (A) WT and *Gsdmd* knockout (*Gsdmd*$^{-/-}$) mice were infected with 10^4 TCID50 of SARS-CoV-2 P21 and examined at 3 dpi for levels of cytokines and chemokines, measured by ELISA of lung homogenates (n=4 mice per genotype). Wilcoxon rank-sum statistical tests were performed.

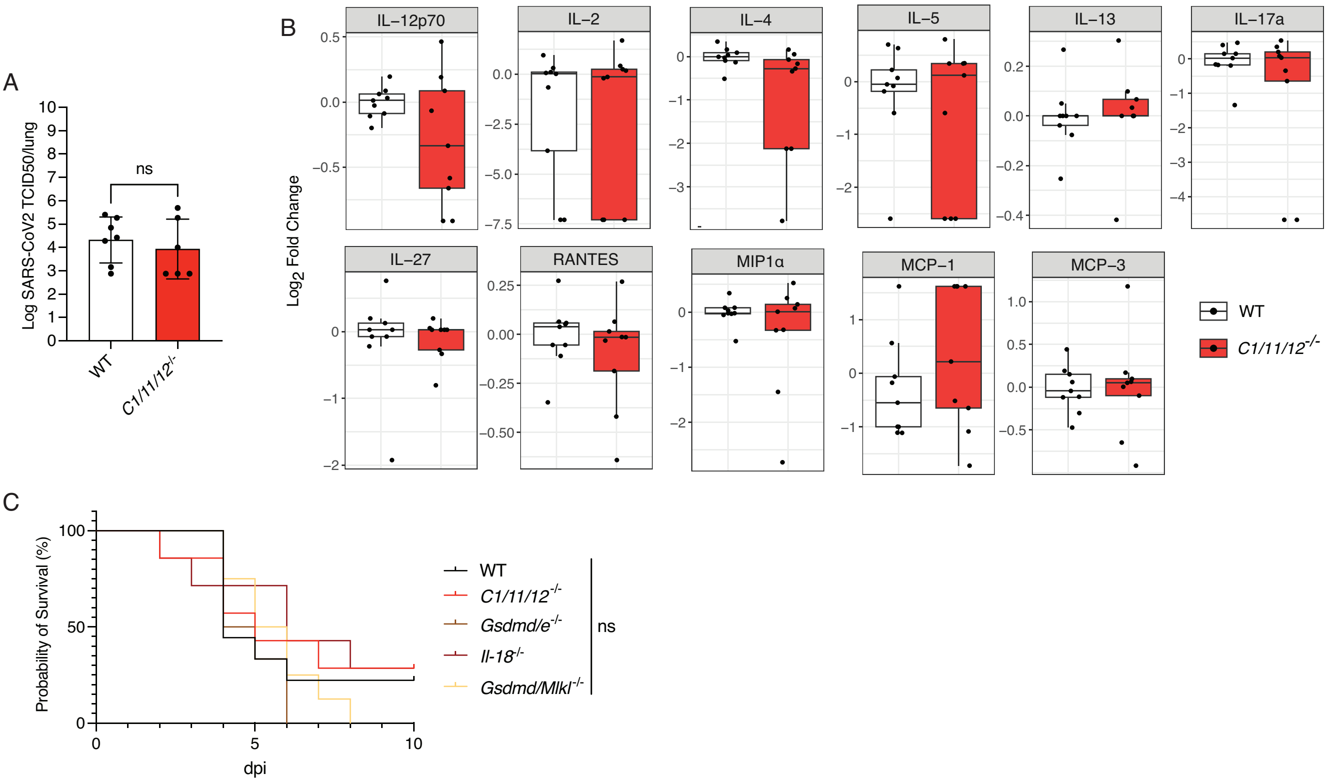

A
B
C
Log SARS-CoV2 TCID50/lung
ns
WT
C1/11/12-/-
Log2 Fold Change
IL−12p70
IL−2
IL−4
IL−5
IL−13
IL−17a
IL−27
RANTES
MIP1α
MCP−1
MCP−3
WT
C1/11/12-/-
Probability of Survival (%)
dpi
WT
C1/11/12-/-
Gsdmd/e-/-
Il-18-/-
Gsdmd/Mlkl-/-
ns

Fig. S3: Caspases-1/-11/-12 do not contribute to SARS-CoV-2-driven disease. (A) WT and *Caspase-1,-11,-12* triple knockout (*C1/11/12*$^{-/-}$) mice were infected with 10^4 TCID50 of SARS-CoV-2 P21 and examined at 6 dpi for viral load by using a TCID50 assay. Data are presented as mean ± SD (n=6-7 mice per group). **(B)** WT and *Caspase-1,-11,-12* triple knockout (*C1/11/12*$^{-/-}$) mice were infected with 10^4 TCID50 of SARS-CoV-2 P21 and examined at 3 dpi for levels of cytokines and chemokines, measured by ELISA of lung homogenates (n=8-9 mice per genotype). **(C)** Aged (>6 month-old) WT and different knockout mice were infected with 10^4 TCID50 of SARS-CoV-2 P21 and monitored for the proportion of mice that became severely ill, reaching predetermined ethical endpoint (n=6-8 mice per genotype). Unpaired two-tailed Student's t test after $\log_{10}$ transformation (A), Wilcoxon rank-sum tests (B) and Log-rank Mantel–Cox statistical tests (C) were performed.

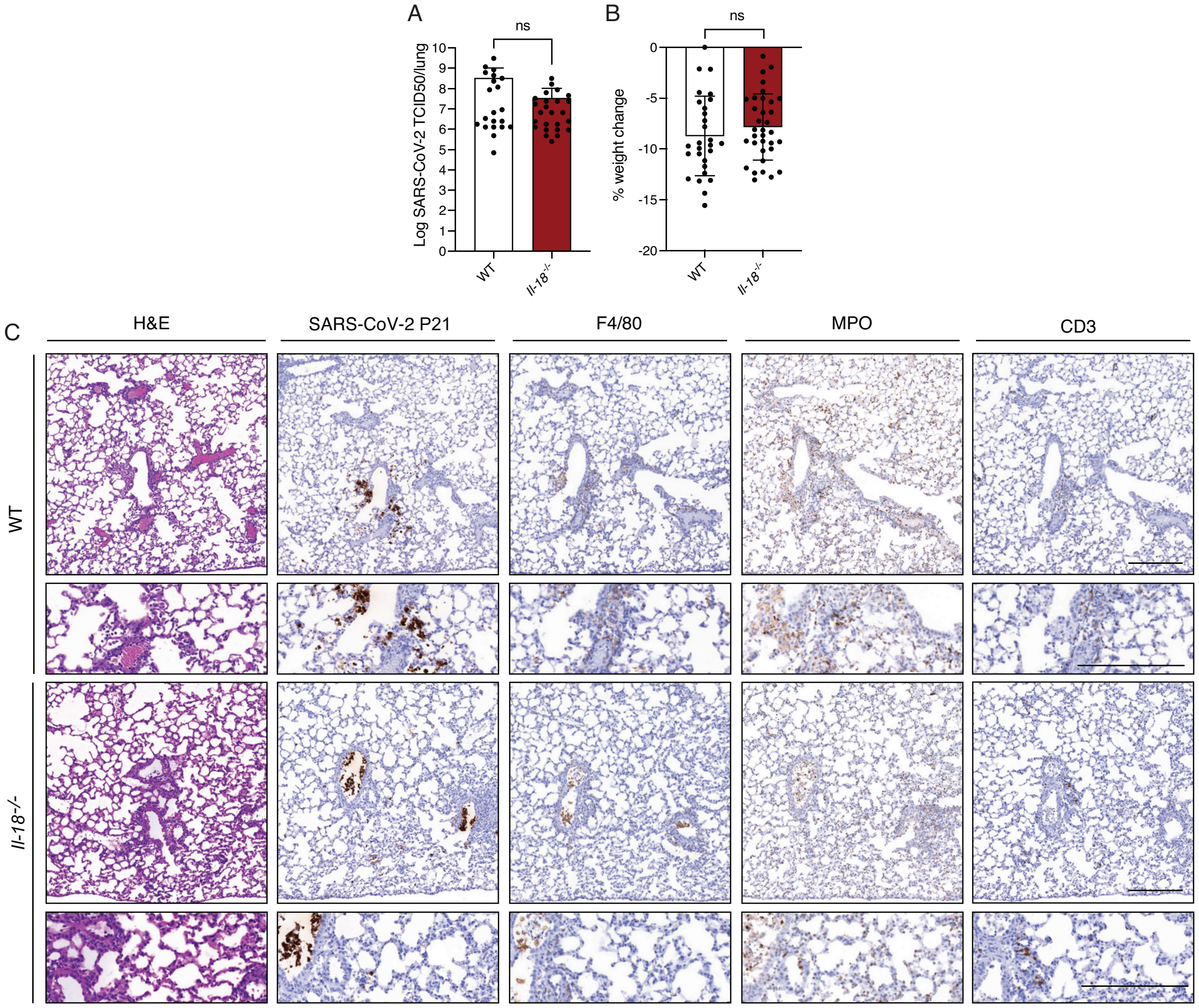
A
ns
Log SARS-CoV-2 TCID50/lung
10
9
8
7
6
5
4
3
2
1
0
WT
Il-18-/-
B
ns
% weight change
0
-5
-10
-15
-20
WT
Il-18-/-
C
H&E
SARS-CoV-2 P21
F4/80
MPO
CD3
WT
Il-18-/-

Fig. S4: *Il-18* knockout mice are not protected from severe SARS-CoV-2 induced disease. (A-*C*) WT and Il-18 knockout (*Il-18*$^{-/-}$) mice were infected with 10^4 TCID50 of SARS-CoV-2 P21 and examined at 3 dpi for **(A)** lung viral burden by using a TCID50 assay and **(B)** percent weight change compared to initial weight (n=17-21 mice per genotype) and **(C)** histological examination of lung pathology. Representative images of haematoxylin and eosin (H&E) and immunohistochemistry (IHC) stained lungs testing for SARS-CoV-2 nucleocapsid, F4/80 (marker of macrophages), MPO (marker of neutrophils) and CD3 (marker of T cells). Histological images are representative of at least 3 animals per genotype. Scale bar = 250 μm. Statistical analyses were performed by unpaired two-tailed Student's t test after $\log_{10}$ transformation (A), unpaired two-tailed Student's t test (B).

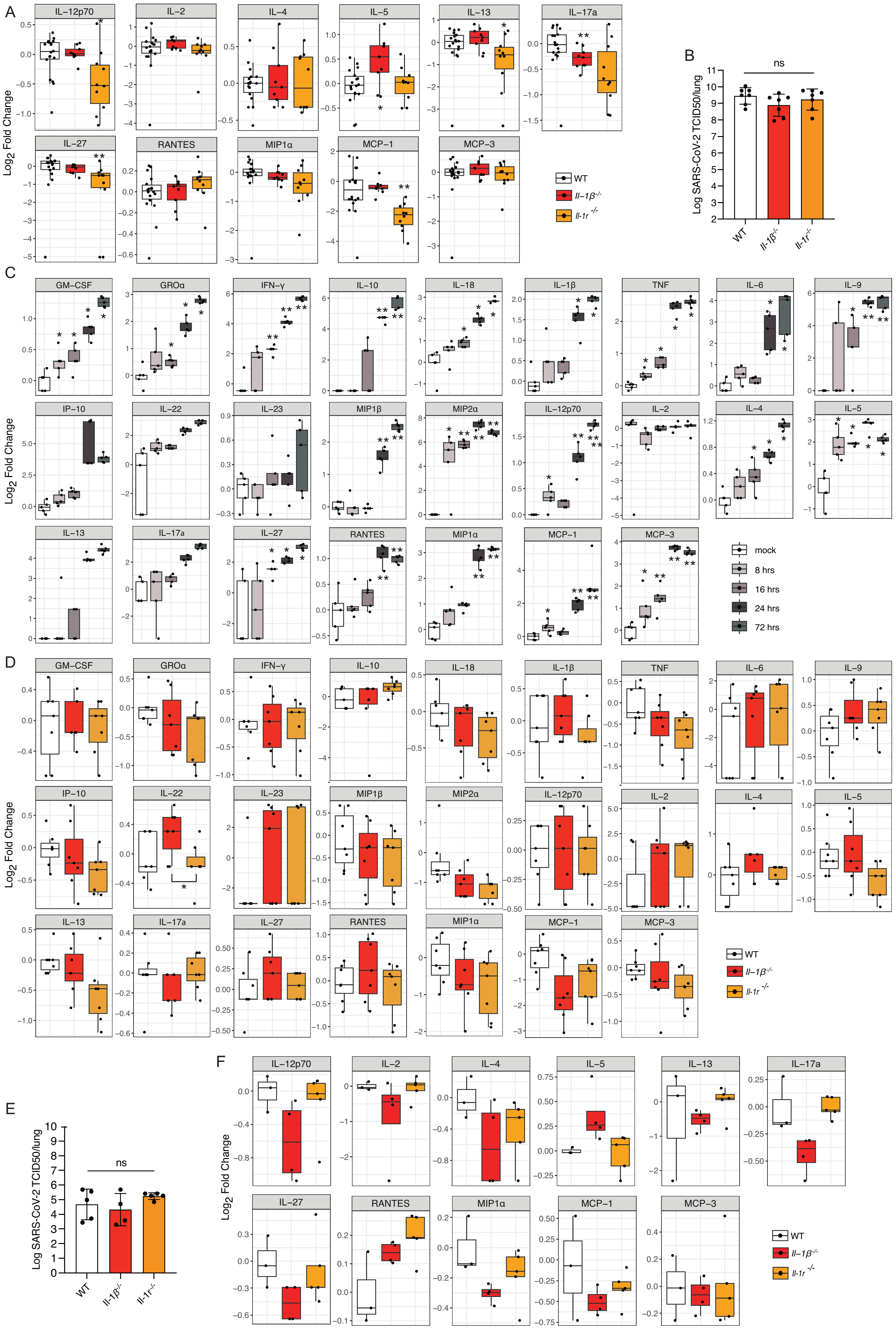
A
IL–12p70
IL–2
IL–4
IL–5
IL–13
IL–17a
IL–27
RANTES
MIP1α
MCP–1
MCP–3
Log2 Fold Change
WT
Il–1β-/-
Il-1r -/-
B
ns
Log SARS-CoV-2 TCID50/lung
WT
Il-1β-/-
Il-1r-/-
C
GM–CSF
GROα
IFN–γ
IL–10
IL–18
IL–1β
TNF
IL–6
IL–9
IP–10
IL–22
IL–23
MIP1β
MIP2α
IL–12p70
IL–2
IL–4
IL–5
IL–13
IL–17a
IL–27
RANTES
MIP1α
MCP–1
MCP–3
Log2 Fold Change
mock
8 hrs
16 hrs
24 hrs
72 hrs
D
GM–CSF
GROα
IFN–γ
IL–10
IL–18
IL–1β
TNF
IL–6
IL–9
IP–10
IL–22
IL–23
MIP1β
MIP2α
IL–12p70
IL–2
IL–4
IL–5
IL–13
IL–17a
IL–27
RANTES
MIP1α
MCP–1
MCP–3
Log2 Fold Change
WT
Il–1β-/-
Il-1r -/-
E
ns
Log SARS-CoV-2 TCID50/lung
WT
Il-1β-/-
Il-1r-/-
F
IL–12p70
IL–2
IL–4
IL–5
IL–13
IL–17a
IL–27
RANTES
MIP1α
MCP–1
MCP–3
Log2 Fold Change
WT
Il–1β-/-
Il-1r -/-

Fig. S5: *Il-1β*$^{-/-}$ and *Il-1r*$^{-/-}$ mice respond differently to SARS-CoV-2 infection. (A) WT, *Il-1β*$^{-/-}$ and *Il-1r*$^{-/-}$ mice were infected with 10^4 TCID50 of SARS-CoV-2 P21 and examined at 3 dpi for levels of cytokines and chemokines, measured by ELISA of lung homogenates (n=8-18 mice per genotype). Mean ± SD are shown. **(B)** WT, *Il-1β*$^{-/-}$ and *Il-1r*$^{-/-}$ mice were infected with SARS-CoV-2 P21 and examined 24 h post-infection for lung viral burden by using a TCID50 assay. **(C)** WT mice were infected with SARS-CoV-2 P21 and examined 8, 16, 24 and 72 h post-infection for 26 cytokines and chemokines via ELISA. Log_2 fold values are shown and they were calculated by normalisation to mock (intranasal administration of DMEM) animals (n=5 mice per group). **(D)** WT, *Il-1β*$^{-/-}$ and *Il-1r*$^{-/-}$ mice were infected with SARS-CoV-2 P21 and examined for levels of cytokines and chemokines at 24 h post-infection by ELISA of lung homogenates (n=6-7 mice per genotype). **(E-F)** WT, *Il-1β*$^{-/-}$ and *Il-1r*$^{-/-}$ mice were infected with SARS-CoV-2 P21 and examined at 6 dpi for **(E)** lung viral burden by using a TCID50 assay and **(F)** levels of cytokines and chemokines at 6 dpi by ELISA of lung homogenates (n=3-4 mice per genotype). Mean ± SD are shown. Statistical analyses were performed: Wilcoxon rank-sum tests (A, C, D, F) and one-way ANOVA after log_{10} transformation (B, E). In (A, D, F), significance is shown relative to WT mice and in (C), significance is shown relative to mock mice (* < 0.05; **p < 0.01).

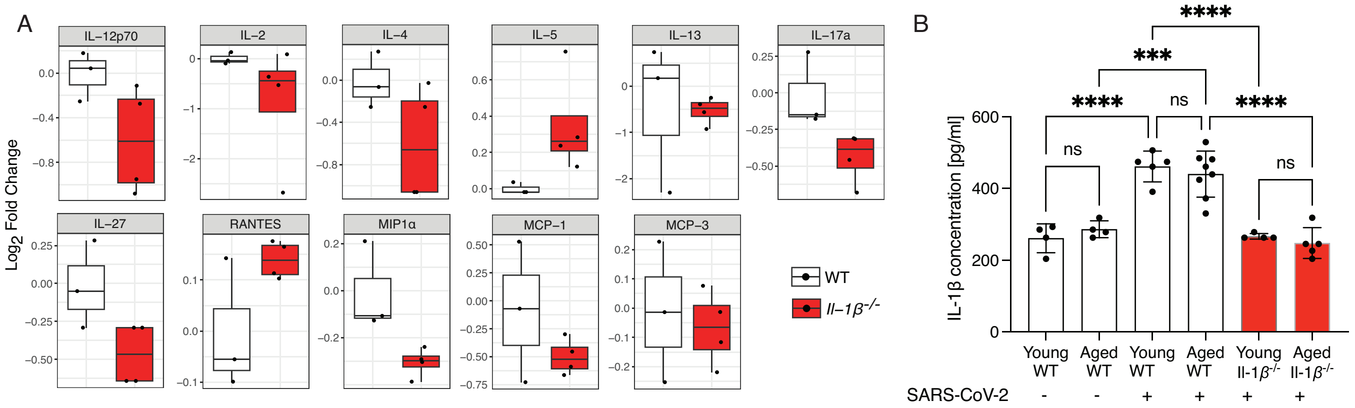
A
Log2 Fold Change
IL–12p70
IL–2
IL–4
IL–5
IL–13
IL–17a
IL–27
RANTES
MIP1α
MCP–1
MCP–3
WT
Il–1β-/-
B
IL-1β concentration [pg/ml]
****
***
****
ns
****
ns
ns
Young WT
Aged WT
Young WT
Aged WT
Young Il-1β-/-
Aged Il-1β-/-
SARS-CoV-2
-
-
+
+
+
+

Fig. S6: Age driven disease severity can be partially ameliorated by removal of IL1-β. (A) Aged (6 month-old) WT and *Il-1β*$^{-/-}$ mice were infected with 10^4 TCID50 of SARS-CoV-2 P21 and examined at 3 dpi for levels of cytokines and chemokines measured by ELISA of lung homogenates (n=3-4 animals per genotype). **(B)** Young (8 week-old) and aged (6 month-old) WT and *Il-1β*$^{-/-}$ mice were either challenged with mock (intranasal administration of DMEM) or infected with 10^4 TCID50 of SARS-CoV-2 P21 and examined at 3 dpi for IL-1β levels *by* ELISA of lung homogenates (n=4-8 animals per group). Wilcoxon rank-sum statistical tests (A) and one-way ANOVA (B) tests were performed.
