# Supplementary figures and images for: IL-1β drives SARS-CoV-2-induced disease independently of the inflammasome and pyroptosis signalling
Source: Cell Death Differ. 2025 Feb 28;32(7):1353–66. doi: 10.1038/s41418-025-01459-x (PMC12284219; doi:10.1038/s41418-025-01459-x)

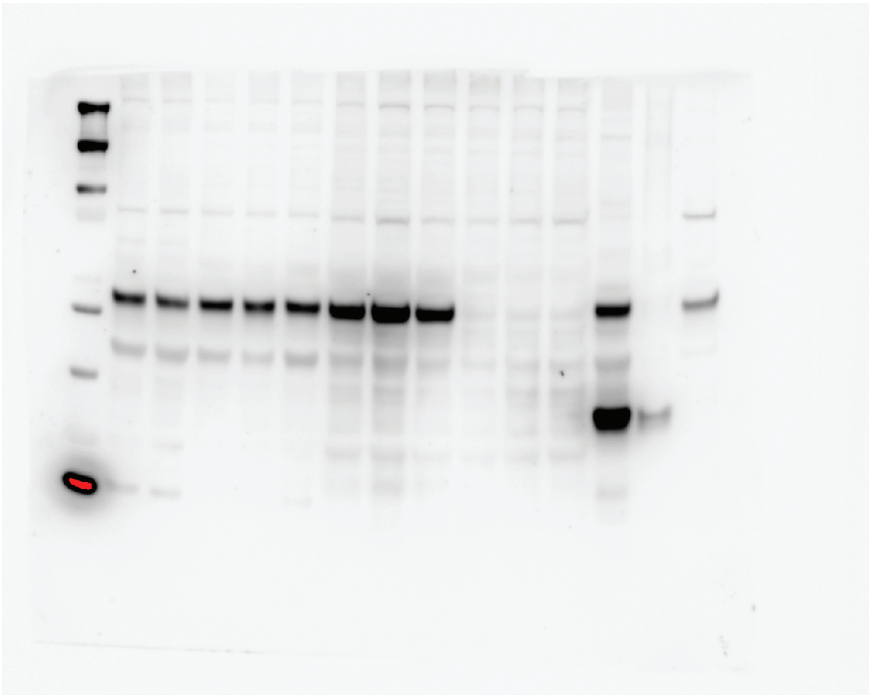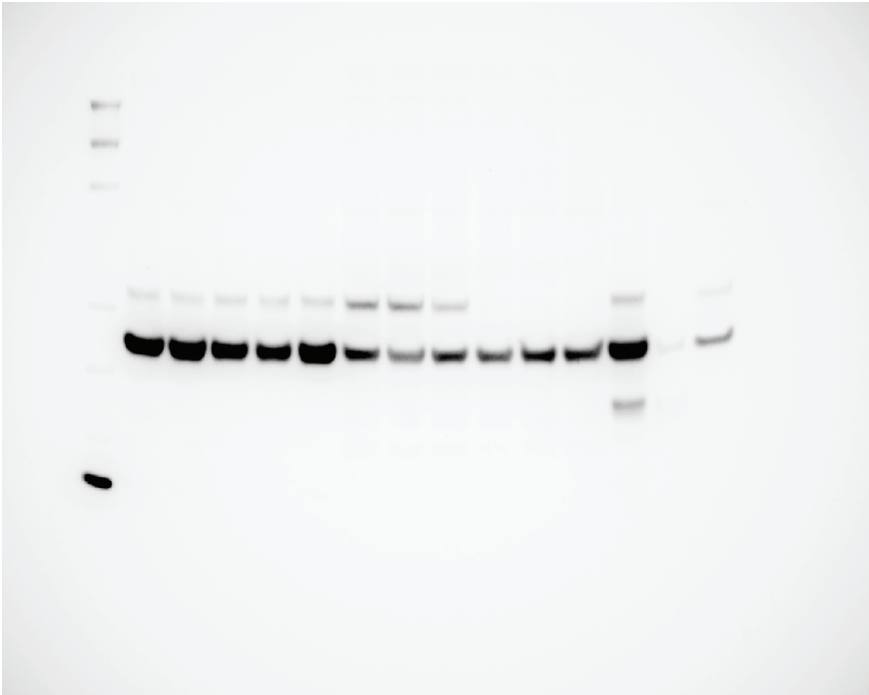

Supplement: Supplementary file 2 — Original Data Files [file 41418_2025_1459_MOESM2_ESM.pdf]
